# Impact of Covid-19 on Referrals to Paediatric Liaison Psychiatry at Children's Health Ireland (CHI) at Crumlin as the Pandemic Moved to Endemic Status

**DOI:** 10.1192/bjo.2024.253

**Published:** 2024-08-01

**Authors:** Bohan Sun, Dimitrios Adamis, Fiona McNicholas

**Affiliations:** 1 School of Medicine and Medical Science, University College Dublin, Dublin, Ireland; 2 Sligo Mental Health Services, Sligo, Ireland; 3 Department of Paediatric Liaison Psychiatry, Children's Health Ireland at Crumlin, Dublin, Ireland; 4 Lucena Clinic Rathgar, St John of God Hospitaller Services, Dublin, Ireland

## Abstract

**Aims:**

Rates of acute mental health presentations in youth were rising pre-pandemic internationally. Longitudinal studies following Covid-19 attest to ongoing deterioration in youth mental health, recognising adverse unintended consequences following public health restrictions.

This study aimed to examine whether the initial reported post-Covid-19 increase in mental health presentations persisted following the reclassification of Covid-19 to endemic status, which was accompanied by removal of most restrictions.

**Methods:**

All referrals to paediatric liaison psychiatry (PLP) between Jan 2018–Dec 2022 in a Dublin tertiary children's hospital were included in the study. An interrupted time series analysis was conducted examining referrals with respect to different phases of Covid-19 and application of public health restrictions.

**Results:**

1,385 referrals to PLP were received over the 5-year study time-period. There was a significant decrease in PLP referrals immediately post Covid-19, following a significant and sustained increase as the pandemic progressed. Public health restriction phases had a unique effect on those presenting with suicidal ideation, with a significant increase in the number of referrals received. There was no effect of restrictions on other clinical profiles.

**Conclusion:**

Increased referrals for youth with mental health difficulties, reported during the Covid-19 pandemic, persisted into the early endemic stage, after Covid-19 public health restriction have ceased. Potential impacts of restrictions on referrals of youth with suicidal ideation require further study. Investment in child and adolescent mental health services remain a priority, and future pandemic responses need to examine unintended consequences of any enforced public health measure.

